# Stepping towards More Intuitive Physical Activity Metrics with Wrist-Worn Accelerometry: Validity of an Open-Source Step-Count Algorithm

**DOI:** 10.3390/s22249984

**Published:** 2022-12-18

**Authors:** Benjamin D. Maylor, Charlotte L. Edwardson, Paddy C. Dempsey, Matthew R. Patterson, Tatiana Plekhanova, Tom Yates, Alex V. Rowlands

**Affiliations:** 1 Assessment of Movement Behaviours Group (AMBer), Leicester Lifestyle and Health Research Group, Diabetes Research Centre, University of Leicester, Leicester LE1 7RH, UK; 2 NIHR Leicester Biomedical Research Centre, Leicester LE5 4PW, UK; 3 MRC Epidemiology Unit, Institute of Metabolic Science, University of Cambridge, Cambridge CB2 1TN, UK; 4 Baker Heart and Diabetes Institute, Melbourne 3004, Australia; 5 The Realtime Building, Clonshaugh Business and Technology Park, Shimmer Research Ltd., D17 H262 Dublin, Ireland

**Keywords:** physical activity, accelerometry, ambulatory measurement, algorithms

## Abstract

Stepping-based targets such as the number of steps per day provide an intuitive and commonly used method of prescribing and self-monitoring physical activity goals. Physical activity surveillance is increasingly being obtained from wrist-worn accelerometers. However, the ability to derive stepping-based metrics from this wear location still lacks validation and open-source methods. This study aimed to assess the concurrent validity of two versions (1. original and 2. optimized) of the Verisense step-count algorithm at estimating step-counts from wrist-worn accelerometry, compared with steps from the thigh-worn activPAL as the comparator. Participants (n = 713), across three datasets, had >24 h continuous concurrent accelerometry wear on the non-dominant wrist and thigh. Compared with activPAL, total daily steps were overestimated by 913 ± 141 (mean bias ± 95% limits of agreement) and 742 ± 150 steps/day with Verisense algorithms 1 and 2, respectively, but moderate-to-vigorous physical activity (MVPA) steps were underestimated by 2207 ± 145 and 1204 ± 103 steps/day in Verisense algorithms 1 and 2, respectively. In summary, the optimized Verisense algorithm was more accurate in detecting total and MVPA steps. Findings highlight the importance of assessing algorithm performance beyond total step count, as not all steps are equal. The optimized Verisense open-source algorithm presents acceptable accuracy for derivation of stepping-based metrics from wrist-worn accelerometry.

## 1. Introduction

Physical activity (PA) of any intensity is associated with reduced all-cause mortality [1,2] and reduced risk of chronic disease, including type 2 diabetes [3] and cardiovascular disease [4]. Current physical activity guidelines for adults recommend ≥150 min/week of moderate-to-vigorous PA (MVPA) for optimal health benefits [5]. Meanwhile, number of steps per day as a way of describing physical activity level is a simple and easily accessible metric [6] and intuitively resonates with the general public to a greater degree than time spent in MVPA [7]. Evidence also suggests that number of steps per day has a similar pattern of association with health outcomes as time in MVPA [2,8,9].

During the past decade there has been an exponential rise in the consumer wearables market, whereby 21% of US adults now own a wearable device with the capability of monitoring physical activity (Pew Research, 2020). A recent systematic review showed that wearing a consumer physical activity device is effective at increasing physical activity [10]. These monitors are primarily worn on the wrist and most focus on the number of steps achieved per day as their main physical activity metric. Furthermore, these metrics are estimated using proprietary algorithms which are not shared with researchers. In addition, research-grade physical activity monitors are increasingly worn at the wrist, e.g., in UK Biobank [11] and the National Health and Nutrition Examination survey in the USA [12]. Recently, in the UK Biobank dataset, del Pozo Cruz and colleagues found that more daily steps were associated with a lower risk of all-cause and cancer mortality for up to 10,000 steps/day [13]. However, other findings have demonstrated benefits to health outcomes such as cardiovascular disease risk markers, cardiovascular events and all-cause mortality are markedly reduced beyond ~8000 steps a day in adults [2,8,14,15]. Apart from the most recent study by del Pozo Cruz et al. [13], the evidence-base relating to step-counts and health has mostly originated from hip- or thigh-worn accelerometers, which, until recently, were the common placement locations for research accelerometry. Thus, there remains a distinct lack of research pertaining to step counts derived from wrist-worn accelerometry. Wrist-worn accelerometers are increasingly being used within large-scale population surveillance research [11,16]. Therefore, being able to generate step-counts is desirable in order to provide clearer physical activity prescription, which can also be self-monitored using consumer wearable devices.

Alongside the growth in use of commercial and research-grade wrist-worn accelerometers, numerous methods exist for deriving step counts from wrist-worn accelerometers, most of which remain proprietary and vary widely in accuracy. Toth et al. [17] reported that when wearing two different devices on each wrist and using different step-detection methods to estimate step counts during one free-living day, the difference in the number of steps between actual steps derived from video-recorded steps and the step-detection methods ranged between −25% to +102%. Recently, Ducharme et al. [18] presented a peak detection method for estimating steps from hip- and wrist-worn accelerometers, showing greater accuracy than existing proprietary algorithms available within the ActiLife software. Furthermore, two different neural network algorithms were trained and tested by Luu et al. [19], and reported a high step-count accuracy compared with video-recorded steps when applied to the open-source Clemson dataset [20]. However, this was assessed in three different activities to simulate different gait patterns in a small sample of 30, thus, its ability to detect steps in free-living conditions remains unknown. The open source Verisense step-count algorithm for wrist accelerometer data [21] is another peak detection algorithm that was validated using the Clemson dataset [20], that has demonstrated accurate detection of steps (mean absolute percent error of <10%) during a controlled 45-minute cardiac rehabilitation session in 22 older adults [22], but had a tendency to underestimate cadence as walking speeds increased [23]. This same algorithm was used in a recent UK Biobank analysis [13]. However, to date there are no data on the performance of this algorithm in a free-living setting. Further, the algorithm has recently been optimized following validation during walking and running to better capture cadence as walking speed increases [23]. Thus, there is a need for detailed robust free-living comparison of the original and optimized Verisense methods, for estimating steps from wrist-worn accelerometry, with valid established methods. This provides an evidence base for use of the algorithm and informs further optimization of algorithms, where warranted. The aim of this study was to assess the concurrent validity of the Verisense step-count algorithm at estimating step-counts from wrist-worn accelerometery in three free-living datasets.

## 2. Materials and Methods

### 2.1. Design and Participants

Data from three trials were used in this analysis to ensure representation of age, PA levels and accelerometer brand.

SMART Work and Life: A total of 756 desk-based workers were recruited from six councils in the UK [24]. To be eligible for this study, participants were required to be ≥18 years of age, work ≥60% full-time equivalent, spend most of their waking day sitting and be able to walk without assistance. Baseline data from this study for both activPAL and Axivity devices were used in this analysis. Ethical approvals from the two lead institutions were obtained prior to the study commencing.

Equivalency: A sample of 56 adults was recruited from two UK universities (University of Leicester and Loughborough University) to wear multiple different brands of accelerometer on the dominant and non-dominant wrists, and the thigh to assess changes in PA metrics between wear location and brands [25]. We used the Axivity data only worn on the non-dominant wrist for this analysis, in addition to the activPAL data. No inclusion/exclusion criteria were specified other than the ability to wear the accelerometer devices during the monitoring period. Ethical approval was obtained from ethics representatives from the University of Leicester prior to the study commencing.

STAND UP: A total of 60 participants aged ≥60 and 65–79 years who engaged in ≤75 min of self-reported vigorous-intensity PA per week and were able to walk unassisted were eligible (Clinical trial number NCT02453204). We used free-living data from both the activPAL and GENEActiv monitors in this analysis. Ethical approval was provided from an NHS Research Ethics Committee (Derby, UK).

### 2.2. Participant Characteristics

Self-reported demographic data were obtained during baseline visits. Participant sex, ethnicity, date of birth (from which age was calculated), height and body mass were collected. Height and weight were used to calculate body mass index (BMI) for descriptive purposes.

### 2.3. Accelerometer Assessment

Wrist-worn accelerometers: The Axivity was worn on the non-dominant wrist in SMART Work and Life and Wrist equivalency studies. The GENEActiv was worn on the non-dominant wrist in STAND UP study. Across all studies, the accelerometers were set up to record at a sampling frequency of 100 Hz and with a dynamic sampling range of ±8 g. Devices were worn continuously for 24 h/day on the non-dominant wrist for 7 days, except for SMART Work and Life which measured behaviors over 8 days.

Thigh-worn accelerometer: The activPAL (model: activPAL3 micro) was used in all three studies, recording accelerations at a sampling frequency of 20 Hz and with a dynamic range of ±2 g. The device was attached to the right thigh and worn for 7 days, except for SMART Work and Life, in which it was worn for 8 days. Participants wore the monitor continuously 24 h/day, only removing it to take part in water-based activities or to re-attach the device with a fresh dressing. Steps per day determined from the activPAL was used to assess concurrent validity of the Verisense algorithm. The activPAL was the reference measure as it has demonstrated high accuracy in detecting step count and stepping cadence during controlled laboratory and free-living conditions [26,27,28]

### 2.4. Deriving Steps from Wrist-Worn Accelerometer Data: Algorithm Conception and Development

The algorithm chosen (henceforth referred to as the Verisense algorithm) for this analysis was based on initial work estimating steps using acceleration patterns from smartphones [29]. This algorithm uses a peak detection method with a set minimum acceleration threshold. Each peak is subsequently assessed for periodicity, similarity, and continuity to filter out artefacts that are not steps [29]. Due to the tendency for a high number of false positives observed when applying this method to wrist-worn accelerometry [21] an additional threshold was included which removed steps if the magnitude of the acceleration peak was not sufficient. Initially, 7776 combinations of thresholds were assessed prior to the best performing combination being published online, referred to as Verisense 1 hereafter [21]. Following preliminary validation in SMART Work and Life data [30], a training subset of 16 participants from the SMART Work and Life dataset, representing participants with good agreement, as well as under- and over-estimation of step counts, was used to optimize the parameter thresholds within the algorithm to reduce bias. Data for these 16 participants were not included in this analysis because the parameter thresholds were optimized specifically for those files. Thus, the sample size for the SMART Work and Life study was reduced from N = 656 to 640. In total, 1152 different parameter threshold combinations were tested on the subset of data. and the combination which balanced performance improvements in under- and over-estimation of step counts was deemed most appropriate to apply to the full dataset. These optimized algorithm parameter thresholds and the Verisense algorithm are referred to as Verisense 2 hereafter (see Table 1 for parameter threshold specifications between both versions of the algorithm and Supplemental material S1 for further information on algorithm parameter optimization).

### 2.5. Accelerometer Data Processing

All devices were initialized for data collection and downloaded, following wear, using the relevant manufacturers’ software. Axivity devices were initialized and raw data downloaded in raw.cwa format using OmGui (OmGui Version 1.0.0.30, Open Movement, Newcastle UK). GENEActivs were initialized, and raw data downloaded in .bin format using GENEActiv PC (version 3.1). The activPAL devices were initialized and raw data downloaded in .csv format using PALanalysis (version 8.11.8.75, PAL Technologies, Glasgow, UK). Raw activPAL data were processed in GGIR to make use of the non-wear detection algorithms within this process to ensure that only days with synonymous 24 h wear for both monitors were included in the analysis. Raw data for the Axivity, GENEActiv and activPAL were processed within open source software R (version 4.1.2, www.cran.r-project.org, accessed on 23 March 2022) using R-package GGIR version 2.6-0 [31]. GGIR is an R-package to process multi-day raw accelerometer data for physical activity and sleep research. Signal processing within GGIR calibrates the raw data using local gravity as a reference [32], identifies periods of non-wear and instances of data clipping due to high acceleration values. Following this, the raw acceleration across three axes were combined to summarize dynamic acceleration as Euclidian Norm minus 1 g (ENMO), expressed in milli-gravitational units (mg), and averaged over 5 s epochs. Parameters used in GGIR are detailed in Supplementary methods S2. We utilized the external function embedding feature present in GGIR to synonymously run the Verisense algorithms 1 and 2 on the raw wrist accelerometer data, with the number of steps detected provided per 5 s epoch. From this we derived step cadence per 5 s epoch to generate the number of steps that were MVPA steps (≥100 steps/min), before collapsing the dataset to daily data.

Following the above procedures, any day of data which resulted in a post-calibration error greater than 10 mg was removed from the analysis. Additionally, only days containing 24 h wear-time from both the wrist and the thigh monitor were included (days which relied on imputation of data from other days to generate 24 h data were removed). The PAL batch was used to generate daily summaries for time spent sitting, standing and stepping, and the number of steps achieved.

### 2.6. Statistical Analysis

Continuous participant characteristics were calculated as mean ± standard deviation (SD) and categorical data as the number (percentage). Bland–Altman validation analysis was used to compare the number of steps and MVPA steps between the activPAL and the Verisense algorithms for each dataset and the datasets combined. Mean bias and limits of agreement (LoA) were generated to describe the level of agreement [33]. All analyses were performed in Rstudio using the Blandr package (version 0.5.1). All values are expressed as mean (95% Confidence intervals), unless stated otherwise. Bland–Altman plots were also generated within R using packages Blandr and ggplot2 (version 3.3.5).

## 3. Results

In total, 713 (86%) participants across the three studies had data from both the wrist accelerometer and activPAL on ≥1 day of 24 h wear. This consisted of 640 (85%), 46 (82%) and 27 (90%) from the SMART Work and Life, Equivalency and STAND UP studies, respectively. Table 2 summarizes the descriptive characteristics of the participants in each study separately and combined. Briefly, participants were aged 44.6 (9.7) years, 71.0% female, 71.2% White European and with a mean BMI of 26.2 (5.9). STAND UP participants were the least active, and Equivalency participants the most active regardless of whether PA was derived from the wrist or thigh accelerometers; though differences between groups were greater when PA was measured at the thigh, compared with the wrist (see Table 2).

**Figure 1:**
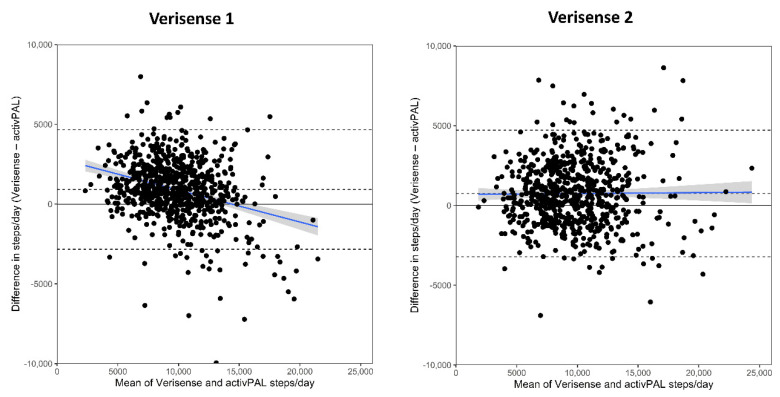
Bland–Altman plot showing the differences in total steps/day derived from the Verisense 1 and Verisense 2 algorithms compared with the activPAL across the mean total steps/day for the overall sample. The blue line depicts mean (95% CI) proportional bias. Dashed black lines depict ± 95% limits of agreement and mean bias. LoA, Limits of Agreement.

**Figure 2:**
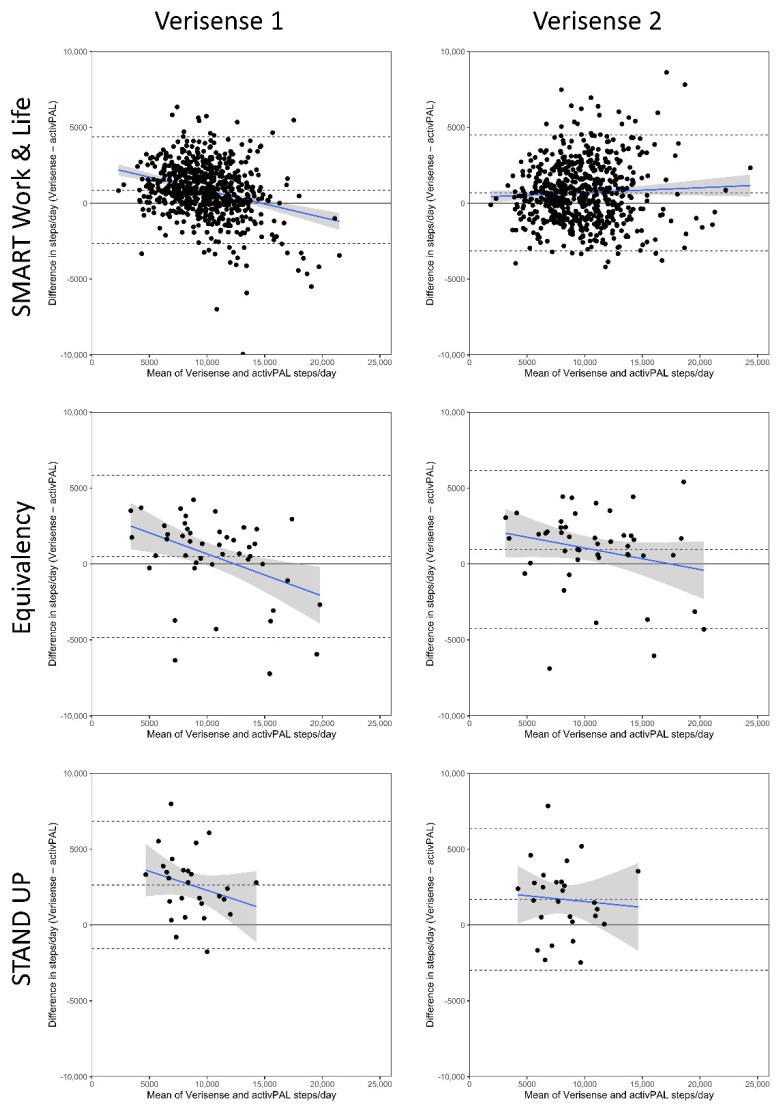
Bland–Altman Plots of total steps/day derived from the Verisense algorithm compared with the activPAL split by study and algorithm version. The blue line depicts mean (95% CI) proportional bias. Dashed black lines depict ± 95% limits of agreement and mean bias. LoA, Limits of Agreement.

The Bland–Altman results for each study, and overall, are shown in Table 3 and Figure 1 (all studies combined) and Figure 2 (separate studies). Datapoints above 0 indicated that the Verisense algorithm overestimated steps, relative to the activPAL, while datapoints below the line indicated that the Verisense algorithm underestimated steps. relative to the activPAL. Results of the original algorithm (Verisense 1) are in the left panel and those for the optimized algorithm (Verisense 2) in the right panel. Across all three samples combined, the Verisense 1 algorithm resulted in a mean overestimation of 913 (772, 1054 (95% limits of agreement, upper and lower dashed lines)) steps/day compared with the activPAL (see Figure 1, left panel). This was equivalent to a 9.7% overestimation. The bias in steps from the Verisense 2 algorithm was lower, with an overestimation of 742 (592, 891) steps a day, equating to a 7.9% overestimation. Similar limits of agreement between the two algorithms were seen (Table 3). There was also a proportional bias observed in the Verisense 1 algorithm, evident in the negative slope of the best-fit line (Figure 1, left panel). This suggested that, as the number of activPAL assessed daily steps increased, the bias reduced and started underestimating steps from ~13,000 steps and above. However, the Verisense 2 algorithm resulted in no proportional bias (horizontal best-fit line) across the range of daily steps (Figure 1, right panel), except for a small proportional bias in the Equivalency dataset (Figure 2, middle right).

When assessing the samples separately, the overestimation of steps was largest in the least active sample, STAND UP, for both the Verisense 1 and Verisense 2 algorithms. despite the mean bias between the Verisense 1 and Verisense 2 being reduced by a third (Table 3). In the most active sample, Equivalency, despite initially having the smallest bias in the Verisense 1 algorithm. of 501 steps/day, in the Verisense 2 algorithm exhibited an increased bias to 942 steps/day within the Equivalency sample, with the limits of agreement remaining similar.

**Figure 3:**
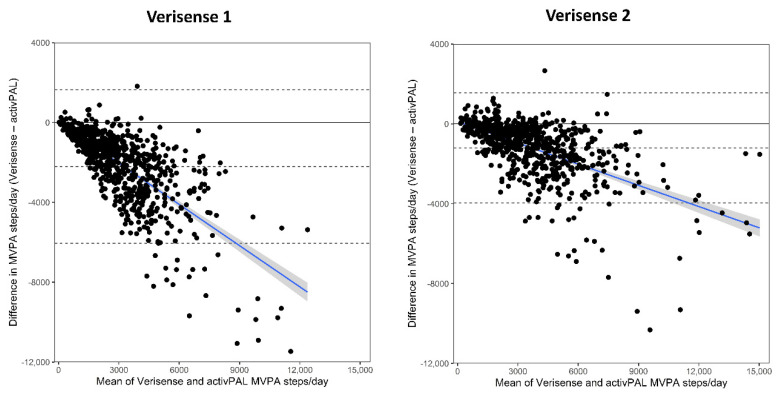
Bland–Altman plot showing the differences in MVPA steps/day derived from the Verisense 1 and Verisense 2 algorithms compared with the activPAL across the mean total steps/day for the overall sample. The blue line depicts mean (95% CI) proportional bias. Dashed black lines depict ± 95% limits of agreement and mean bias. LoA, Limits of Agreement.

**Figure 4:**
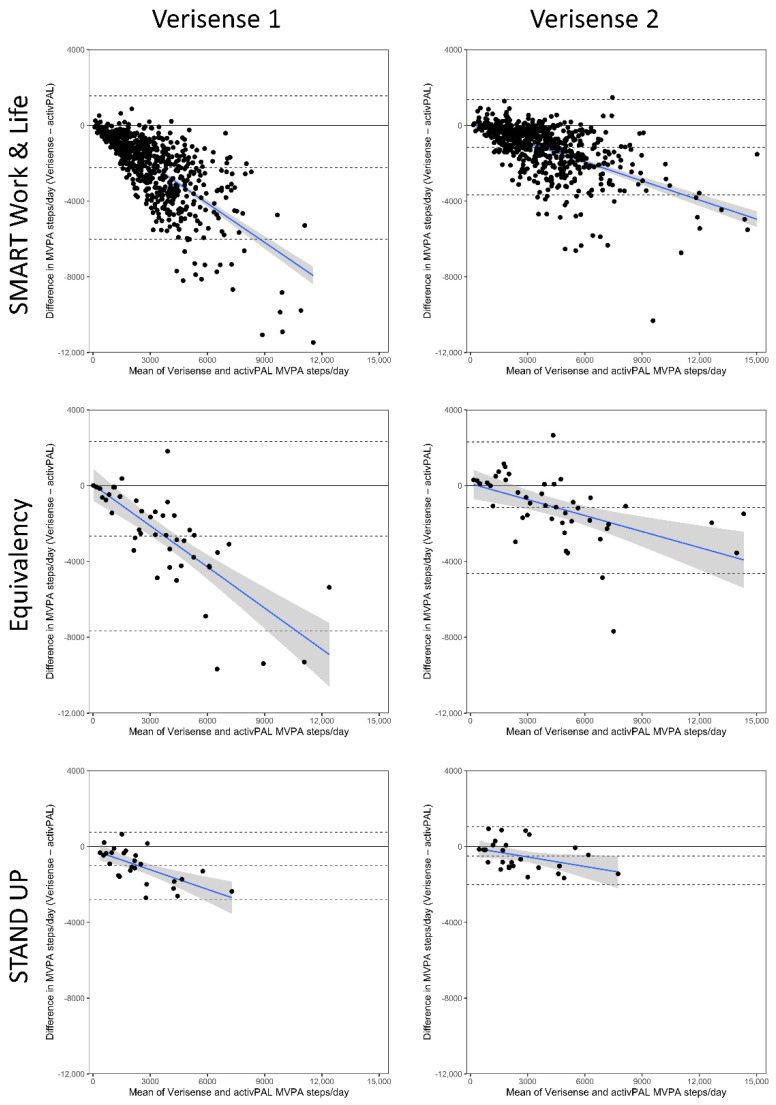
Bland–Altman Plots of MVPA steps/day derived from the Verisense algorithm compared with the activPAL split by study and algorithm version. The blue line depicts mean (95% CI) proportional bias. Dashed black lines depict ± 95% limits of agreement and mean bias. LoA, Limits of Agreement.

The Bland–Altman results for the number of steps at a MVPA intensity are shown in Table 4 and Figure 3 (all studies combined) and Figure 4 (separate studies). Across all three samples combined the number of MVPA steps derived from the Verisense 1 algorithm was 2207 (2062, 2351) steps/day lower compared with the number of MVPA steps/day derived from the activPAL (Table 4). The number of MVPA steps derived from the Verisense 2 algorithm resulted in a smaller underestimation of 1204 (1101, 1307) steps/day compared with MVPA step/s day derived from the activPAL (Figure 3, right panel compared with the left panel). The relative change in the underestimation of MVPA steps/day between Verisense 1 and Verisense 2 algorithms were broadly similar, with reductions of 46%, 48% and 51% in the Equivalency, STAND Up and SMART Work and Life datasets, respectively. All three datasets, combined (Figure 3) and individually (Figure 4), displayed a proportional bias, such that, as the number of MVPA steps/day increased, the underestimation of MVPA steps, compared with the activPAL, increased (negative sloped best-fit line). This proportional bias improved slightly in the Verisense 2 algorithm, though remained evident.

## 4. Discussion

This study examined the performance of an open-source algorithm to estimate daily stepping-based metrics from wrist-worn accelerometer data, before and after optimization of the parameter thresholds on a subset of data to improve estimation accuracy. Our results showed that the step count from a wrist-worn accelerometer resulted in mean overestimation of 913 (9.7%) steps/day, compared with the activPAL worn on the thigh. However, with the optimized algorithm, the overestimation reduced to 742 (7.9%) steps/day, whilst greatly reducing the proportional bias observed. In contrast to the overestimation of total steps, MVPA steps (>100 steps/min) were underestimated. This is consistent with the underestimation of cadence observed during higher walking speeds and running [23]. Importantly, the optimized version of the Verisense algorithm (Verisense 2) improved the accuracy, with a shift from underestimating MVPA steps by 2207 steps/day to underestimating MVPA steps by 1204 steps/day. This showed that the updated parameter thresholds performed better at estimating both step volume and intensity outcomes. However, the bias that remained is still noteworthy and should be considered when comparing steps using this algorithm to steps from the activPAL.

Our finding that the total daily step count estimates were higher when derived from the wrist compared with the thigh is consistent with similar research investigating differences in behavioral metrics based on accelerometer wear location. Using activities of daily living, previous analyses have shown that estimates of step count were ~1200 steps/day higher when derived from consumer-grade wrist-worn devices. compared with a pedometer worn at the waist [34,35] and direct observation [36]. When comparing ActiGraph accelerometers worn synonymously on the waist and wrist during 7 days of free-living, Tudor-Locke et al. [37] reported the device worn on the wrist estimated 2500 more steps/day compared with that on the waist. Our analysis also resulted in the overestimation of steps/day, likely due to the higher rate of false positives observed during activities which involve predominantly the use of arms and hands, for example, washing the dishes, preparing food or using a computer. This overestimation of steps/day was evident despite MVPA steps being underestimated. The relative balance of MVPA and other activities in each sample could explain why the SMART Work and Life and STAND UP samples, which were less active in terms of activPAL steps, reported higher bias than the more active Equivalency participants. Indeed, Ducharme et al. [18] reported a 2.8× higher prediction error in step counts from the wrist compared with the waist, describing the difficulty of deriving steps from the wrist location, due to erroneous classification of superfluous wrist movements that are common in free-living behavior. In terms of magnitude of overestimation, Toth et al. [17] assessed the estimation of step counts from a research-grade accelerometer worn on the non-dominant wrist using three different proprietary algorithms compared with video-recorded steps, as the criterion measure. They reported a −28% to +195% mean absolute percent error. The mean absolute percent error for Verisense 2 was 20.9%, which is modest in comparison, supporting its use in future research. An algorithm including use of further wearable sensors, e.g., GPS or accelerometers on additional body locations may lead to improved accuracy. However, it would not be possible to apply such an algorithm to the large datasets worldwide that currently deploy wrist-worn research-grade accelerometers.

When comparing the number of MVPA steps estimated by both versions of the Verisense algorithm, we saw a marked improvement in the underestimation between algorithms compared with the comparator, equivalent to ~50% reduction in the bias in the Verisense 2 algorithm compared with Verisense 1. As the algorithm was optimized to reduce the total step count bias, it is possible that the parameters could be altered further to perform better in detecting MVPA steps in the future. However, the underestimation of MVPA steps within each sample did improve substantially following optimization This is consistent with improved performance of the optimized algorithm observed during walking and running [23]. Understanding performance of the algorithm for different ranges of cadence is important as cadence may be an important factor in the prevention of adverse health outcomes, such as type 2 diabetes [3,38]. Until recently, the majority of studies reported limited associations of step cadence with all-cause mortality when adjusting for total daily steps [8,15]. However, the UK Biobank analysis by del Pozo Cruz et al. [13] did find that higher stepping cadence for the peak 30 minutes of the day was associated with lower incident CVD and all-cause mortality outcomes, independent of total daily steps. Despite disparities in the literature about its relevance for health, assessing an algorithm’s ability to perform accurately in distinguishing between steps of different intensities in free-living data, while minimising false positives and false negatives, is an important factor in algorithm development. However, there has been limited attention to this in the literature to date.

### Strengths and Limitations

The main strengths of this study were the large sample size with concurrent wrist and thigh accelerometry free-living data across three diverse datasets covering young, middle-aged and older adults. Despite the sample mostly consisting of office workers, there was a wide range of physical activity within the overall sample which assisted in the optimization of parameters for the Verisense 2 algorithm, evidenced by the reduction in total and MVPA stepping bias, and, in particular, removing the proportional bias observed for total step counts in Verisense 1. An additional strength was that the algorithm is open-access [21] and works directly as an external function within the open-source R package GGIR, making it immediately accessible to other researchers for application to their datasets without the need for specialist software. Further, recent research shows that the algorithm performed similarly for three widely used brands of research-grade accelerometer (GENEActiv, ActiGraph and Axivity) worn on either wrist [23]. A limitation in this study was the lack of criterion measure. The activPAL has shown high accuracy when measuring steps during free-living activities and simulated activities of daily living [26,39]. However, it is worth noting that there may be some accuracy lost during particularly low walking speeds, where steps below ~40 steps/min are not detected [39,40]. Numerous health-related research using steps used the activPAL to determine steps during free living [8,41,42]. Due to the free-living nature of the data collected, it was not feasible to directly observe steps on such a large sample.

## 5. Conclusions

In conclusion, this study found that an optimized version of the open-source Verisense step-count algorithm was more accurate than the original version in detecting total and MVPA steps in free-living data. compared to step count from a thigh-mounted activPAL. These findings highlight the importance of assessing algorithm performance beyond total step count, as not all steps are equal, when investigating stepping metrics with markers of health. The refined Verisense open-source algorithm presents acceptable accuracy for derivation of stepping-based metrics from wrist-worn accelerometry.

## Figures and Tables

**Table 1:** Threshold changes between Verisense algorithms.

Threshold Description	Parameter Unit	Verisense 1	Verisense 2
Peak detection threshold	Samples	3	4
Minimum periodicity threshold	Samples	5	4
Maximum periodicity threshold	Samples	15	20
Similarity threshold	G	−0.5	−1.0
Window size of continuity	Samples	4	4
Number threshold of continuity	Number of windows	4	4
Variance threshold for motion recognition	Variance of G	0.001	0.01
Magnitude threshold	G	1.2	1.25

**Table 2:** Participant characteristics for each study.

Characteristic	SMART Work and Life	Wrist Equivalency	STAND UP	Overall
*N*	640	46	27	713
Age (years)	44.8 (10.3)	24.6 (4.5)	73.3 (4.2)	44.6 (9.7)
Sex, *n* female	463 (72.3%)	29 (63.0%)	14 (51.9%)	506 (71.0%)
Ethnicity, *n* White British	455 (71.1%)	39 (84.8%)	14 (51.9%)	508 (71.2%)
BMI (kg/m^2^)	26.4 (6.1)	23.2 (3.8)	26.4 (3.9)	26.2 (5.9)
Sitting time (min/day)	603.6 (94.8)	589.3 (130.0)	557.2 (116.0)	601.0 (98.5)
Standing time (min/day)	233.6 (75.6)	216.1 (76.1)	300.0 (85.4)	235.1 (77.1)
Stepping time (min/day)	110.9 (34.0)	117.8 (52.9)	114.9 (34.7)	111.5 (35.5)
Average acceleration (mg)	27.6 (7.9)	31.1 (9.6)	25.1 (4.9)	27.7 (8.0)
Intensity gradient	−2.54 (0.23)	−2.38 (0.33)	−2.75 (0.20)	−2.55 (0.24)

BMI, Body mass index; values are expressed as mean ± SD or n (%), unless stated otherwise. The intensity gradient describes the negative curvilinear relationship between physical activity intensity and the duration of time spent in that intensity during a 24 h day. A more negative gradient (lower value) represents less time spent in higher intensity activities.

**Table 3:** Agreement between steps derived by the activPAL and steps derived by the Verisense algorithms applied to wrist accelerometer data.

Outcome	SMART Work and Life	Equivalency	STAND UP	Overall
N	640	46	27	713
N valid days	4.9 (4.7, 5.0)	2.1 (1.8, 2.4)	3.9 (3.2, 4.6)	4.7 (4.5, 4.8)
ActivPAL steps	9369 (9117, 9622)	10,345.3 (8924, 11,767)	7282.7 (6214, 8352)	9352.2 (9103, 9601)
Verisense original algorithm (1)				
Verisense steps	10,239 (10,025, 10,454)	10,829 (9723, 11,935)	9922 (9052, 10,792)	10,265 (10,058, 10,472)
Mean difference to activPAL	+870 (730, 1009)	+501 (−311, 1313)	+2639 (1789, 3490)	+913 (772, 1054)
95% LoA’s	−2648, 4388	−4860, 5861	−1573, 6852	−2837, 4663
Verisense refined algorithm (2)				
Verisense steps	10,056 (99,796, 10,316)	11,269 (10,017, 12,521)	8975 (7969, 9981)	10,093 (9844, 10,344)
Mean difference to activPAL	+687 (535, 839)	+942 (150, 1733)	+1692 (749, 2635)	+742 (592, 891)
95% LoA’s	−3139, 4513	−4280, 6164	−2982, 6366	−3235, 4718

*N* valid days, number of days which contained 24 h wear for both accelerometers; LoA, Limits of Agreement. Values are expressed as mean (95% CI) steps per day unless stated otherwise.

**Table 4:** Agreement between MVPA steps derived by the activPAL and MVPA steps derived by the Verisense algorithms applied to wrist accelerometer data.

Outcome	SMART Work and Life	Equivalency	STAND UP	Overall
N	640	46	27	713
N valid days	4.9 (4.7, 5.0)	2.1 (1.8, 2.4)	3.9 (3.2, 4.6)	4.7 (4.5, 4.8)
ActivPAL MVPA steps	4309 (4102, 4516)	5214 (4092, 6336)	2913 (2110, 3716)	4306 (4104, 4508)
Verisense original algorithm (1)				
Verisense MVPA steps	2085 (1979, 2192)	2426 (1851, 3001)	1893 (1345, 2442)	2099 (1995, 2204)
Mean difference to activPAL	−2224 (−2074, −2374)	−2788 (−2055, −3521)	−1019 (−662, −1377)	−2207 (−2062, −2351)
95% LoA’s	−6010, +1562	−7571, +1995	−2791, +752	−6055, +1641
Verisense refined algorithm (2)				
Verisense MVPA steps	3149 (2999, 3299)	3935 (3065, 4805)	2418 (1767, 3070)	3098 (2954, 3243)
Mean difference to activPAL	−1160 (−1061, −1260)	−1279 (−785, −1773)	−494 (−186, −803)	−1204 (−1101, −1307)
95% LoA’s	−3684, +1363	−4503, +1945	−2022, +1033	−3954, +1547

*N* valid days, number of days which contained 24 h wear for both accelerometers; LoA, Limits of Agreement; MVPA steps, moderate to vigorous steps (100 steps/min). Values are expressed as mean (95% CI) Moderate to vigorous steps per day unless stated otherwise.

## Data Availability

The data that support the findings of this study are not openly available due to their containing information that could compromise research participant privacy/consent. Requests for participant-level quantitative data and statistical codes should be made to the corresponding author. Data requests are put forward to members of the original trial management team who release data on a case-by-case basis.

## Supplementary Materials

The following supporting information can be downloaded at: https://www.mdpi.com/article/10.3390/s22249984/s1, Supplementary methods S1: Algorithm Parameter Optimization; S2: GGIR Parameters.
