# Supplementary material for: Stepping towards More Intuitive Physical Activity Metrics with Wrist-Worn Accelerometry: Validity of an Open-Source Step-Count Algorithm
Source: Sensors (Basel). 2022 Dec 18;22(24):9984. doi: 10.3390/s22249984 (PMC9786909; doi:10.3390/s22249984)
Supplement: Supplementary file 1 [file sensors-22-09984-s001.zip › sensors-2059890-supplementary.pdf]

Supplemental Methods 1: Algorithm Parameter Optimization

Adapted from Rowlands et al [23]:
A large (~650) dataset [30] consisting of participants wearing a tri-axial accelerometer on the wrist and activPAL on the thigh was used. Step count was obtained from the wrist-worn accelerometer using the Verisense Step Count Algorithm [21] in GGIR and from the thigh-worn activPAL (Maylor et al., 2022). The Verisense steps algorithm is based off a method presented in Gu et al. [29] with an additional magnitude threshold parameter added. It was found that the Verisense step count differed from the ActivPAL step count on many of the tests.

A process was undertaken to investigate the possibility of matching the Verisense step count to the ActivPAL step count. To do this, a wide range of threshold parameters were chosen for the Verisense step count and a grid search approach was taken on a subset of the original dataset. The subset of data consisted of 16 participants in total, where the error between the original Verisense step count and ActivPAL step count was distributed over the range of overestimating by 3, 000 steps per day to underestimating by less than 3, 000 steps per day. Since the step count difference in the full dataset was evenly distributed, the subset of even disturbed step count difference data was used to tune parameters on the Verisense algorithm. The range of Verisense algorithm parameters tested are listed in Table 1, with the original algorithm parameters being shown in bold. All combinations of variables were tested, resulting in 1, 152 parameter combinations being tested.

Table 1 - Verisense step algorithm threshold that were tested. Each combination of thresholds was tested on a subset of data. Original algorithm parameters are shown in bold.

**Threshold**	**Symbol**	**Tested Values**
Peak detection threshold	k	**3**, 4
Minimum periodicity threshold	t_min	4, **5**
Maximum periodicity threshold	t_max	**15**, 20, 25
Similarity threshold	sim	-1.0, **-0.5**, -0.1
Window size of continuity	M	3, **4**, 5
Number threshold of continuity	N	**4**, 6
Variance threshold for motion recognition	var	**0.001**, 0.01
Magnitude threshold	mag	1.0, **1.2**, 1.25, 1.3, 1.4

For each combination of thresholds, step count was obtained for each day for each participant on the subset of 16 participants. Mean absolute error for each threshold combination was obtained by first calculating the absolute difference in steps for each day and then taking the mean of all values for each threshold combination (Equation 1)

$$MAE = \frac{Absolute(StepsVerisense - StepsTarget)}{number\ of\ observations} \qquad \text{(Equation 1)}$$

Where StepsVerisense are the number of steps calculated using the Verisense algorithm and some combination of thresholds, StepsTarget are the target steps and number of observations are the

total number of days compared for each combination of thresholds. Figure 1 shows the resultant MAE values for each threshold combination tested.

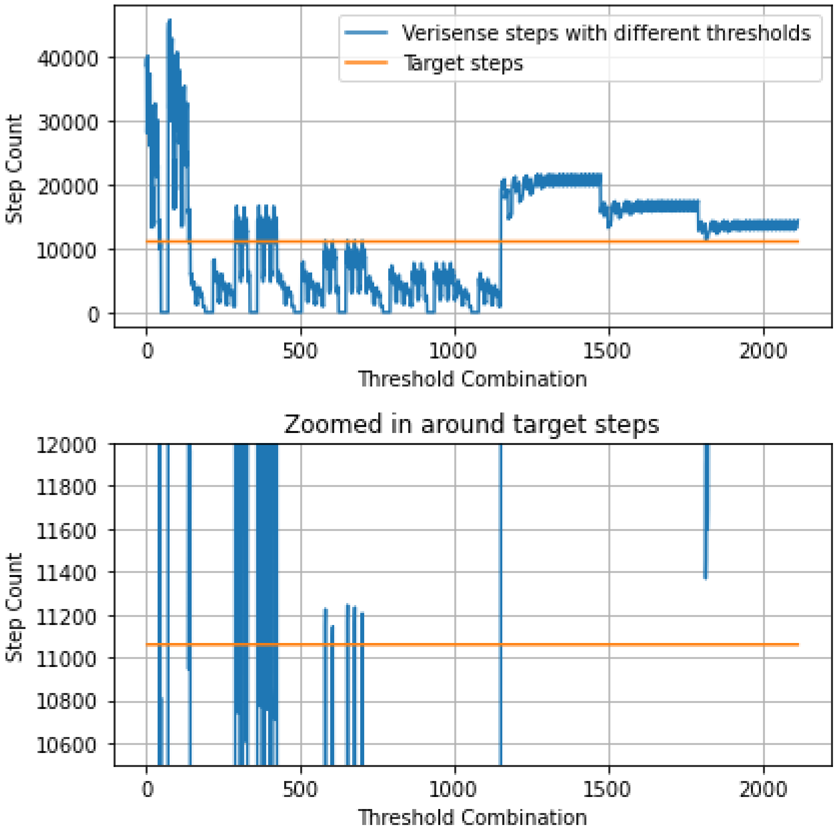

Figure 1 - Total step count for each threshold combination, with the target steps shown on the horizontal orange line. Threshold combinations with a final step count that are close to the target step count are of intertest to optimize algorithm performance.

It was found that the threshold combination that resulted with the lowest MAE on the full data subset was prioritising performance on tests that were originally underestimating step count, while showing poor performance on tests that were originally over estimating step count (cyan line in Figure 2). The contrast of this is the orange line, which shows a parameter set that optimised performance on the tests that were originally over estimating step count, while showing poor performance on tests that were originally underestimating step count. A balanced approach was taken such that the final parameter set was chosen as the lowest total MAE with MAE on the over-estimating tests under 5, 000 steps and MAE on the under-estimating steps under 5, 000 steps as well. The results of this parameter set are shown on the green line in Figure 2.

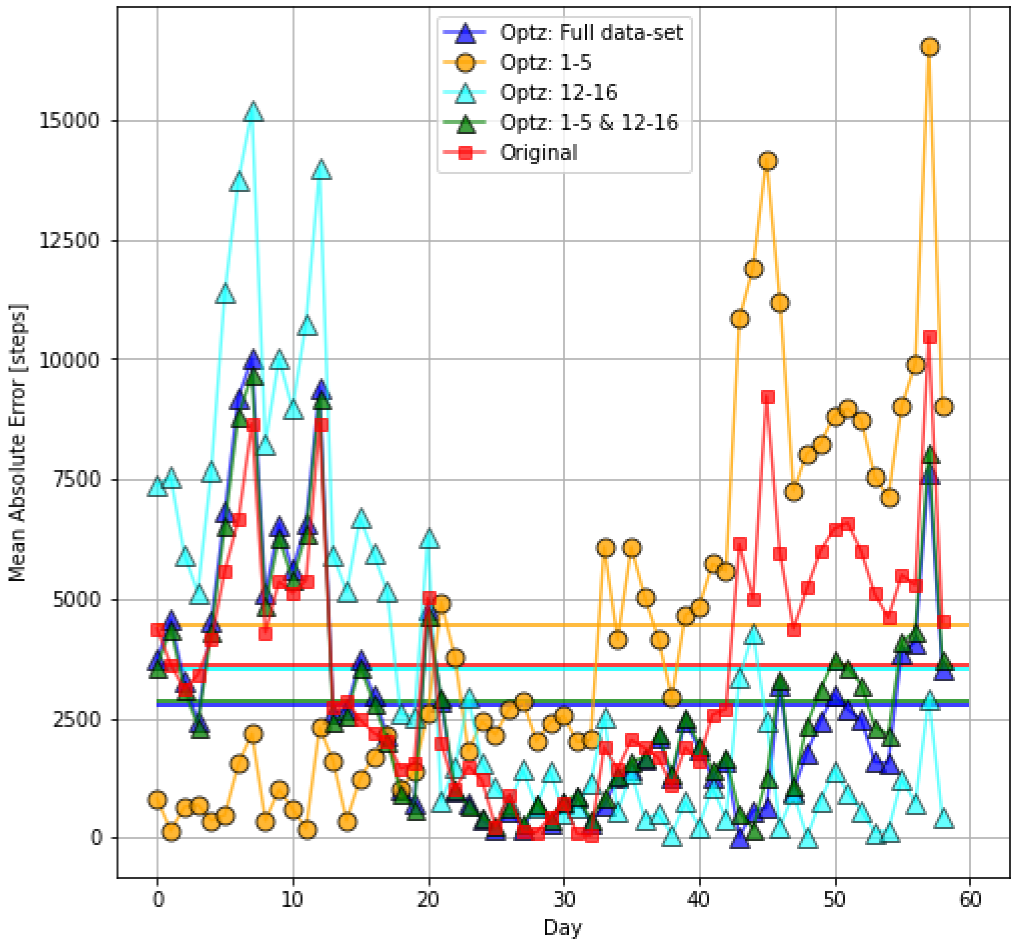

Figure 2 shows the MAE on each day (from 16 participants) in the data subset of various combinations of optimizations.

Table 2 - Original and optimised thresholds along with MAE values.

**Threshold**	**Symbol**	**Original**	**Optimised**
Peak detection threshold	k	3	4
Minimum periodicity threshold	t_min	5	4
Maximum periodicity threshold	t_max	15	20
Similarity threshold	sim	-0.5	-1.0
Window size of continuity	M	4	4
Number threshold of continuity	N	4	4
Variance threshold for motion recognition	var	0.001	.01
Magnitude threshold	mag	1.2	1.25
MAE		3567.2	2837.8

Supplemental Methods 2: GGIR parameters

Parameter	Value	Context
R_version	R version 4.1.2 (2021-11-01)	not applicable
GGIRversion	2.6-0	General parameters
do.report	2	General parameters
mode	c(1, 2)	General parameters
studyname	Verisense	General parameters
qwindow	c(0, 24)	params_247
qlevels	c()	params_247
qwindow_dateformat	%d-%m-%Y	params_247
ilevels	c()	params_247
IVIS_windowsize_minutes	60	params_247
IVIS_epochsize_seconds	c()	params_247
IVIS.activity.metric	2	params_247
qM5L5	c()	params_247
MX.ig.min.dur	10	params_247
M5L5res	10	params_247
winhr	5	params_247
iglevels	c(seq(0, 4000, 25),8000)	params_247
LUXthresholds	c(0, 100, 500, 1000, 3000, 5000, 10000)	params_247
LUX_cal_constant	c()	params_247
LUX_cal_exponent	c()	params_247
LUX_day_segments	c()	params_247
window.summary.size	10	params_247
L5M5window	c(0, 24)	params_247
includedaycrit	16	params_cleaning
ndayswindow	7	params_cleaning
selectdaysfile	c()	params_cleaning
strategy	1	params_cleaning
maxdur	9	params_cleaning
hrs.del.start	0	params_cleaning
hrs.del.end	0	params_cleaning
includedaycrit.part5	0.666666667	params_cleaning
excludefirstlast.part5	FALSE	params_cleaning
TimeSegments2ZeroFile	c()	params_cleaning
do.imp	TRUE	params_cleaning
data_cleaning_file	c()	params_cleaning
minimum_MM_length.part5	23	params_cleaning
excludefirstlast	FALSE	params_cleaning
includenightcrit	16	params_cleaning
excludefirst.part4	FALSE	params_cleaning
excludelast.part4	FALSE	params_cleaning
max_calendar_days	0	params_cleaning
